# 人长链非编码RNA基因SPRY4-ITl对肺癌细胞A549侵袭和迁移能力的影响

**DOI:** 10.3779/j.issn.1009-3419.2015.08.07

**Published:** 2015-08-20

**Authors:** 松 柴, 文婷 安, 丽丽 郭, 乐 姜, 志嵩 高, 书军 李

**Affiliations:** 050000 石家庄，河北医科大学第二医院肿瘤中心 Cancer Center, The Second Hospital of Hebei Medical University, Shijiazhuang 050000, China

**Keywords:** 肺肿瘤, 侵袭, 迁移, Lung neoplasms, Invasion, Migration

## Abstract

**背景与目的:**

人长链非编码RNA基因的异常表达与多种肿瘤有关。本研究旨在探讨人长链非编码RNA基因SPRY4-ITl对肺癌细胞A549侵袭和迁移能力的影响及机制。

**方法:**

将SPRY4-ITl转染入肺癌A549细胞系，采用RT-PCR方法检测重组质粒在细胞中表达水平比较；采用MTT和Transwell检测A549细胞侵袭和迁移能力的变化，采用Western blot方法检测SPRY4-ITl对MMP-2和MMP-9蛋白的影响。

**结果:**

转染pcDNA3.1-SPRY4-ITl细胞划痕两侧细胞间距较转染pcDNA3.1细胞明显变窄，其穿膜细胞数为（207 ± 34）个/视野，相比对照组明显增多（*P* < 0.05），且转染pcDNA3.1-SPRY4-ITl细胞中的基质金属蛋白酶（matrix metalloproteinase, MMP）-2及MMP-9蛋白表达较转染空质粒组均有升高。

**结论:**

在肺癌A549细胞中过表达SPRY4-IT1可增强细胞的侵袭、迁移能力，且细胞内MMP-2及MMP-9表达升高，提示SPRY4-IT1可能通过调控MMP影响肺癌的侵袭和转移能力。

## Abstract (en)

**Background and objective:**

The abnormal expression of human long chain non encoding RNA gene is related to many kinds of tumors. The aim of this study is to investigate the expression of long non-coding RNA maternally expressed gene 3 (SPRY4-IT1) in lung cancer (A549) cells, and to observe the effect of SPRY4-IT1 on the invasion and migration of A549 cells.

**Methods:**

The levels of SPRY4-IT1 in A549 was detected by real-time PCR. The effects of SPRY4-IT1 on the invasion and migration of A549 cell were analyzed by MTT and Transwell assay. The expression of matrix metalloproteinase (MMP) family proteins was determined by Western blot.

**Results:**

The invasion and migration of A549 cells were increased after SPRY4-IT1 over-expression. The cell spaces were narrower after SPRY4-IT1 over-expression in the wound healing assay. Transwell assays showed that the numbers of transmembrane A549 cells were higher in SPRY4-IT1 over-expression group than control group (*P* < 0.05). Meanwhile, over-expression of SPRY4-IT1 reduced the expression of matrix metalloproteinase (MMP)-2 and MMP-9.

**Conclusion:**

Over-expression of SPRY4-IT1 enhanced the invasion and migration of A549 cells. MMP-2 and MMP-9 might play an important role in this regulation.

长链非编码RNA（long non-coding RNA, lnc RNA）是一类长度大于200个核苷酸的非编码RNA，在人类全基因组中有20万-40万种不同的lnc RNA。研究^[1]^表明lncNA可以通过多种方式影响编码基因的表达，在肿瘤的发生发展过程中起调控作用。SPRY4内含子转录本1（SPRY4 intronic transcript 1, SPRY4-IT1）（基因号AK024556）是转录自SPRY4基因的第二个内含子的长度为687个核苷酸的未剪接的、多聚腺苷酸化的转录产物，最初在脂肪组织中得以鉴定，且过表达SPRY4-ITl在黑色素瘤中具有促进细胞生长、迁移和侵袭的作用^[2]^，然而其在肺癌细胞中的作用尚未报道。本研究拟探讨SPRY4-ITl对肺癌细胞A549侵袭和迁移能力的影响，并初步探索可能的作用机制。

## 1 材料和方法

### 1.1 材料

人肺癌细胞系A549、95C、95D、Calu-3、H125、H1299、H1975、SPC-A1由河北医科大学第二医院肿瘤中心提供；抗基质金属蛋白酶（matrix metalloproteinase, MMP）-2抗体、抗MMP-9抗体均购自武汉博士德生物有限公司；Transwell（8 μm孔径）小室及Matrigel胶购自北京乐博生物科技有限公司，实验中所用引物由广州锐博生物有限公司设计并合成，检测SPRY4-IT1在肺癌A549细胞中的表达，RT-PCR上游引物为5’-AGCCACATAAATTCAGCAGA-3’，下游引物为5’-CGATGTAGTAGGATTCCTTT-CA-3’，GAPDH上游引物为: 5’-GACTCATGACCACAGTCCATGC-3’，GAPDH下游引物为: 5’-AGAGGCA-GGGATGATGTTCTG-3’。

### 1.2 基因全长序列克隆

根据GenBank数据库SPRY4-ITl标准序列NR-002196.1设计引物，上游引物5’-TAAGCTTGTAGAGATGGGGGTTTCATCCTGTTGG-3’，下游引物5’-ACTCGAGAAAGACTCCCTTTCCTTAAGCAG ATTCAC-3’，循环条件：93 ℃预变性1 min，93 ℃变性30 s，65 ℃退火30 s，72 ℃延伸2 min，25个循环，72 ℃终末延伸5 min，产物用1%琼脂糖凝胶电泳分离扩增产物并进行产物纯化回收，将产物连接T载体后，转化DH5α感受态细胞，通过蓝白斑筛选，挑取白色克隆，扩增后进行酶切鉴定，同时送测序分析证实扩增片段的正确性。

### 1.3 构建真核表达载体

双酶切pcDNA3.1载体和插入正确片段的T质粒，分别纯化回收目的片段。连接线性化pcDNA3.1载体和目的片段，转化DH5α感受态细胞，进行菌液PCR鉴定，并通过测序分析得到插入片段阳性克隆，命名为pcDNA3.1-SPRY4-ITl表达载体。用无内毒素试剂盒大量制备质粒。

### 1.4 细胞培养和细胞转染^[3]^

使用含10%小牛血清的RPMI-1640培养液，在37 ℃、含5%CO_2_的培养箱内培养实验中用到的肺癌细胞系。细胞生长融合度达到50%时，用lipo 2000进行细胞转染，分为转染空载体pcDNA3.1组和转染pcDNA3.1-SPRY4-ITl表达载体组。

### 1.5 RT-PCR检测肺癌细胞中SPRY4-Itl的表达

培养肺癌细胞，细胞生长融合度打70%时收集细胞，通过Trizol法提取细胞总RNA，逆转录合成cDNA，应用上述RT-PCR引物扩增SPRY4-ITl，ABI7300检测并分析各细胞中SPRY4-IT1的水平。

### 1.6 细胞划痕迁移实验

将A549细胞以每孔5×10^5^个接种于6孔板中，按照上述实验分组转染48 h后，细胞融合度达到90%以上，用无菌移液枪头部划线，置于37 ℃、5%CO_2_的细胞培养箱中培养24 h后光学显微镜下拍照，随即取5个视野，测量划痕两侧细胞间距取平均值，每组实验设3个复孔，实验重复3次。

### 1.7 Transwell侵袭实验

将A549细胞以每孔5×10^5^个接种于6孔板中，细胞生长融合度为60%时按照上述实验分组转染空载体和pcDNA3.1-SPRY4-ITl过表达质粒，转染24 h后收集转染后的各组细胞，重悬于无血清RPMI-1640培养液，以每孔5×10^4^个细胞加入预铺好Matrigel的Transwell小室上室内，下室加入500 μL完全培养液，置于37 ℃、5%CO_2_细胞培养箱中培养24 h后多聚甲醛固定，0.05%结晶紫染色，400倍光学显微镜下计数穿膜细胞数，随即取10个视野，取平均值，每组实验设3个复孔，实验重复3次。

### 1.8 免疫印迹实验^[4]^

收集细胞划痕实验后的各组细胞，用胰酶消化并收集，用含有蛋白酶抑制剂的RIPA裂解细胞，置于冰上，每5分钟震摇细胞1次，共裂解30 min，15, 000转/分在4℃离心机上离心15 min，取上清，即为提取的细胞蛋白质。通过BCA法检测蛋白浓度，水浴变性蛋白，加入5×SDS-PAGE上样缓冲液，加样等量蛋白的蛋白裂解液，用10%的SDS-PAGE胶进行电泳分离，电转印到尼龙膜上，用含5%脱脂牛奶的PBS缓冲液室温封闭1小时，一抗用1:1, 000稀释的抗人MMP-2、MMP-9单克隆抗体4℃过夜孵育，用PBS-Tween洗膜，室温每次15 min，共3次；印迹膜用1:3, 000的抗鼠IgG偶联的辣根过氧化物酶作为二抗，室温孵育1h，用PBS-Tween洗膜，室温每次15 min，共3次。用增强型化学发光液检测试剂盒化学发光显色。检测细胞中MMP-2、MMP-9的表达，以β-actin作为内参照，结果进行灰度值分析并通过统计学检验，实验重复3次。

### 1.9 统计学处理

采用SPSS 13.0统计分析软件，计量资料采用均数±标准差（Mean±SD）表示，组间比较采用*t*检验，以*P* < 0.05为差异有统计学意义。

## 2 结果

### 2.1 pcDNA3.1-SPRY4-ITl真核表达载体构建

以cDNA为模板，用引物扩增目的片段，得到与预期片段大小相符的特异性片段（图 1A）。切胶纯化回收目的片段，并与pGEM-Teasy载体连接，插入目的片段后，通过酶切鉴定和测序分析证实插入片段的正确性（图 1B、图 1C）。将目的片段SPRY4-ITl与pGEM-Teasy载体连接后的重组体经*Bam*H Ⅰ+*Hin*d Ⅲ双酶切，与相同双酶切线性化的pcDNA3.1载体连接，得到完整的pcDNA3.1-SPRY4-ITl表达载体，通过*Bam*H Ⅰ+*Hin*d Ⅲ酶切鉴定和测序分析（图 1D、图 1E），确认载体构建成功。将重组质粒pcDNA3.1-SPRY4-ITl和空质粒pcDNA3.1转染A549细胞后，检测细胞中SPRY4-ITl表达，结果提示转染重组质粒pcDNA3.1-SPRY4-ITl的细胞中SPRY4-ITl表达量是转染空质粒的32.7倍（图 2）。

**1:**
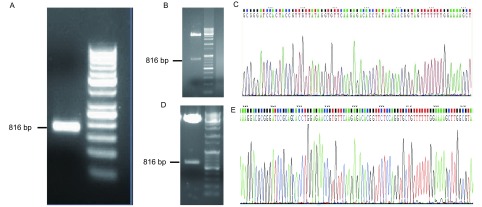
pcDNA3.1-SPRY4-ITl真核表达载体构建情况。A：目的片段扩增产物；B：目的片段与T载体连接后酶切鉴定结果；C：目的片段与T载体连接后测序结果；D：目的片段与pcDNA3.1载体连接后酶切鉴定结果；E：目的片段与pcDNA3.1载体连接后测序结果。 pcDNA3.1-SPRY4-ITl eukaryotic expression vector construction. A: Amplification products of target fragment; B: Enzyme digestion identification results of target fragment and T vector; C: Sequencing results of target fragment and T vector; D: Enzyme digestion identification results of target fragment and pcDNA3.1 vector; E: Sequencing results of target fragment and pcDNA3.1 vector.

**2:**
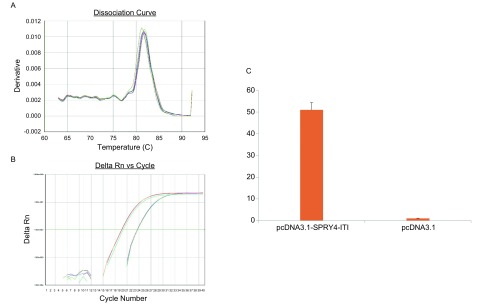
将重组质粒pcDNA3.1-SPRY4-ITl和空质粒pcDNA3.1转染A549细胞后，检测细胞中SPRY4-ITl表达。A：目的基因溶解曲线；B：目的基因扩增曲线；C：SPRY4-ITl在细胞中表达水平比较。 The expression of SPRY4-ITl in pcDNA3.1-SPRY4-ITl and pcDNA3.1 A549 cells. A:Dissolution curve of target gene; B: Amplification curve of target gene; C: The expression of SPRY4-ITl in different cells.

### 2.2 SPRY4-ITl对细胞迁移能力的影响

将A549细胞接种于6孔板，转染pcDNA3.1-SPRY4-ITl和空载体pcDNA3.1，24 h后结果显示转染pcDNA3.1-SPRY4-ITl细胞划痕两侧细胞间距较转染pcDNA3.1细胞明显变窄（图 3），提示A549细胞转染pcDNA3.1-SPRY4-ITl后细胞的迁移能力增强。

**3:**
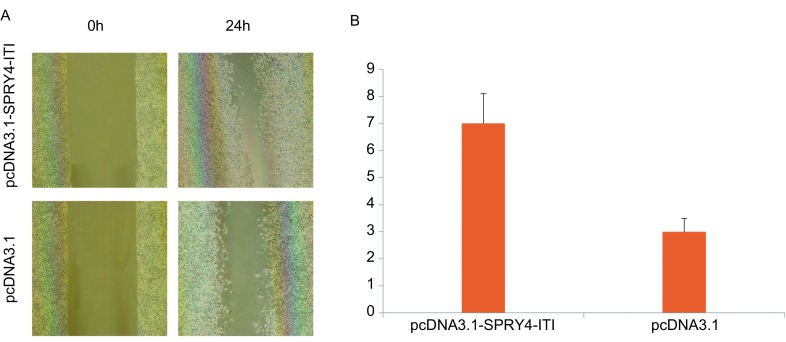
细胞划痕实验结果。A：培养24 h后两组细胞迁移结果的比较（×400）；B：24 h后两组细胞迁移距离比较。 Cell scratch test results. A: Cell migration results of two groups (×400); B: Cell migration spaces of two groups.

### 2.3 SPRY4-ITl对细胞侵袭能力的影响

A549细胞转染pcDNA3.1-SPRY4-ITl后其穿膜细胞数为（207 ± 34）个/视野，对照组为（83±15）个/视野，组间差异有统计学意义（*P* < 0.05）（图 4）。表明A549细胞转染pcDNA3.1-SPRY4-ITl后细胞的侵袭能力增强。

**4:**
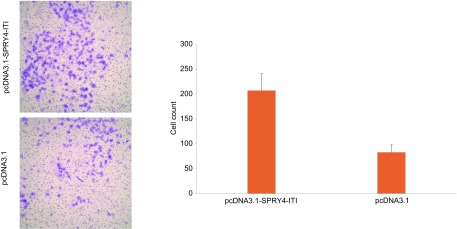
细胞Transwell小室结果。A：显微镜下两组细胞穿过Transwell小室基底膜结果（×400）；B：两组细胞穿过基底膜细胞数目比较。 Transwell assay results. A: Cells through the basement membrane results of two groups (×400); B: The numbers of transmembrane A549 cells of two groups.

### 2.4 SPRY4-ITl对MMP-2和MMP-9蛋白的影响

转染pcDNA3.1-SPRY4-ITl细胞中的MMP-2及MMP-9蛋白表达较转染空质粒组均有升高（图 5）。

**5:**
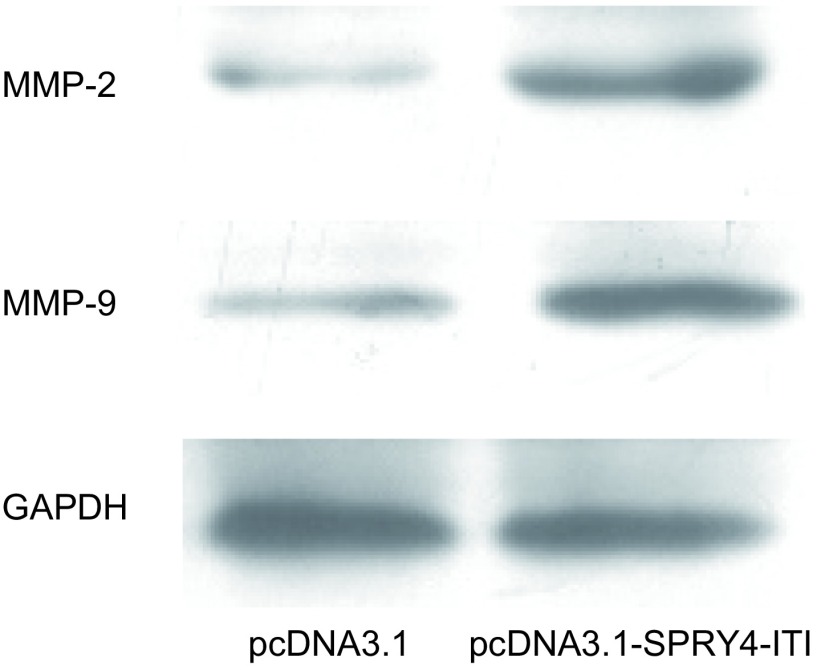
过表达SPRY4-IT1对MMP-2和MMP-9的影响 Effect of overexpression of SPRY4-IT1 on matrix metalloproteinase (MMP)-2 and MMP-9

## 3 讨论

在人类基因组中除了编码基因外，同时包含大量的非编码RNAs（non-coding RNAs, ncRNAs）^[5]^。ncRNAs虽不具备编码蛋白质功能，但能通过不同方式调控编码基因的表达和功能，lncRNA与多种类型的肿瘤发生发展有着密切联系，已成为近年来的研究热点之一^[1]^。这类ncRNAs既可以通过影响蛋白翻译发挥转录后调控，也可以通过影响基因的转录活性及蛋白降解等多种途径发挥功能。

SPRY4-IT1是由SPRY4基因的一个内含子转录而来，其内部含有多个二级发卡结构，已有研究表明SPRY4-IT1在黑色素瘤细胞中过表达，并且参与调控黑色素瘤细胞的凋亡与迁移^[2]^。有关SPRY4-IT1在肺癌中的研究尚未见报道，为了更深入地探讨SPRY4-IT1基因在肺癌细胞中的作用，本研究克隆了SPRY4-IT1基因，并构建了SPRY4-IT1真核表达载体，用脂质体介导转染A549细胞，发现原本在A549细胞中表达量很低的SPRY4-IT1，在转染pcDNA3.1-SPRY4-ITl后，表达量升高了25倍（与转染空载体相比较），能够满足进一步的实验需要。

肺癌细胞最重要的恶性行为即具有高度的侵袭转移能力^[6-8]^，因此我们在肺癌A549细胞中转染pcDNA3.1-SPRY4-IT1重组质粒，观察SPRY4-IT1对肺癌细胞迁移、侵袭能力的影响。我们的研究发现，转染pcDNA3.1-SPRY4-ITl细胞划痕两侧细胞间距较转染pcDNA3.1细胞明显变窄，A549细胞转染pcDNA3.1-SPRY4-ITl后其穿膜细胞数为（207 ± 34）个/视野，对照组为（83±15）个/视野，组间差异有统计学意义（*P* < 0.05），可见通过细胞划痕运动实验和Transwell侵袭实验均证实过表达SPRY4-IT1后可以明显增加A549细胞的侵袭、迁移能力，提示SPRY4-IT1有可能与肺癌细胞的侵袭迁移相关。MMP-2和MMP-9是目前公认的调控肿瘤侵袭转移的关键因子^[9-11]^，为了进一步研究SPRY4-IT1对肺癌A549细胞侵袭迁移能力改变的作用机制，我们通过Western blot检测其中MMP-2及MMP-9的表达，发现在肺癌A549细胞中过表达SPRY4-IT1可以显著增加MMP-2和MMP-9蛋白的表达。

综上所述，在肺癌A549细胞中过表达SPRY4-IT1可增强细胞的侵袭、迁移能力；同时过表达SPRY4-IT1后MMP-2及MMP-9呈升高趋势，提示SPRY4-IT1可能通过对调控MMP影响肺癌的侵袭转移能力。可见SPRY4-IT1在肺癌的侵袭转移中发挥重要作用，为肺癌的临床治疗提供了新的药物靶点及监测指标。
